# Peroxiredoxin 6 Attenuates Alloxan-Induced Type 1 Diabetes Mellitus in Mice and Cytokine-Induced Cytotoxicity in RIN-m5F Beta Cells

**DOI:** 10.1155/2020/7523892

**Published:** 2020-08-25

**Authors:** Elena G. Novoselova, Olga V. Glushkova, Sergey M. Lunin, Maxim O. Khrenov, Svetlana B. Parfenyuk, Tatyana V. Novoselova, Mars G. Sharapov, Vladimir I. Novoselov, Evgeny E. Fesenko

**Affiliations:** Institute of Cell Biophysics of the Russian Academy of Sciences, PSCBR RAS, Institutskaya Str. 3, 142290 Pushchino, Moscow Region, Russia

## Abstract

Type 1 diabetes is associated with the destruction of pancreatic beta cells, which is mediated via an autoimmune mechanism and consequent inflammatory processes. In this article, we describe a beneficial effect of peroxiredoxin 6 (PRDX6) in a type 1 diabetes mouse model. The main idea of this study was based on the well-known data that oxidative stress plays an important role in pathogenesis of diabetes and its associated complications. We hypothesised that PRDX6, which is well known for its various biological functions, including antioxidant activity, may provide an antidiabetic effect. It was shown that PRDX6 prevented hyperglycemia, lowered the mortality rate, restored the plasma cytokine profile, reversed the splenic cell apoptosis, and reduced the *β* cell destruction in Langerhans islets in mice with a severe form of alloxan-induced diabetes. In addition, PRDX6 protected rat insulinoma RIN-m5F *β* cells, cultured with TNF-*α* and IL-1*β*, against the cytokine-induced cytotoxicity and reduced the apoptotic cell death and production of ROS. Signal transduction studies showed that PRDX6 prevented the activation of NF-*κ*B and c-Jun N-terminal kinase signaling cascades in RIN-m5F *β* cells cultured with cytokines. In conclusion, there is a prospect for therapeutic application of PRDX6 to delay or even prevent *β* cell apoptosis in type 1 diabetes.

## 1. Introduction

Insulin-dependent diabetes mellitus, or type 1 diabetes (T1D), is a multifactorial disease, in which autoimmune factors play a key role. Clinical symptoms of T1D manifest themselves when most of insulin-producing pancreatic beta cells have already died because of the activation of autoreactive T lymphocytes. The massive death of insulin-producing *β* cells, which is caused by cytotoxic T lymphocytes migrating into the pancreas, leads to the accumulation of glucose in the blood, and patients with T1D need regular administration of insulin for the rest of their lives. Despite the administration of insulin, T1D causes serious inflammatory complications in many systems and organs, including the cardiovascular system [1], kidneys [2], and eyes [3]. It is known that in diabetes, large blood vessels are especially severely damaged; therefore, mortality from stroke and heart attack is three times higher among patients with diabetes than in the rest of the population.

Currently, most of the studies on the pathogenesis of T1D mellitus focus on pancreatic *β* cells, which are targets of the autoimmune attack. Meanwhile, the disease is usually caused by oxidative stress and imbalances in the immune system, which are related to autoaggressive clones of T lymphocytes [4, 5]. During autoimmune inflammatory reactions, proinflammatory cytokines, including interleukin- (IL-) 1*β*, tumor necrosis factor-alpha (TNF-*α*), and interferon- (IFN-) *γ*, are released into the environment of *β* cells by activated T cells and macrophages, causing *β* cell dysfunction and death [6, 7]. Usually, a proinflammatory response protects the mammalian organism from foreign pathogens and maintains the integrity of tissues and cellular systems. However, a defective proinflammatory response may cause the opposite effect, increasing the risk of autoimmune pathologies, which include T1D [6].

It is known that human T1D is sometimes linked with altered genes providing susceptibility to diabetes [8]. However, studies on identical twins with familial diabetes showed that only approximately half of them develop diabetes [9], confirming an important role of environmental factors, such as dietary factors during infancy, vaccination, and others [10], in the risk of development of T1D [11]. T1D susceptibility involves a complex interplay between genetic and environmental factors and has historically been attributed to adaptive immunity, although there is now increasing support for a role of innate inflammation [12].

Oxidative stress has been proven to play a key role in the pathogenesis of diabetes and related complications [13], and there is evidence that antioxidants, mainly low-molecular-weight natural and synthetic substances, may be useful for the treatment of various pathologies associated with diabetes mellitus [14, 15]. Meanwhile, there are many reasons to believe that antioxidant enzymes can be more effective in neutralising reactive oxygen species (ROS) than low-molecular-weight antioxidants. Previously, we have shown the therapeutic effects of a recombinant peroxiredoxin 6 (PRDX6) in various pathologies associated with inflammation and oxidative stress, such as intestinal hypoxia/reperfusion [16]. We believe that PRDX6 may be effective as an agent that suppresses the level of oxidative stress in diabetes mellitus. Indeed, it was shown that pancreatic *β* cells contain lower levels of antioxidant enzymes, such as SOD, catalase, and GPX, than do other mammalian tissues [17]. Therefore, these cells are more sensitive to the damaging effects of ROS. Because of this deficiency of endogenous antioxidant enzymes in *β* cells, there is an increasing interest in the use of external proteins with antioxidant activities to protect pancreatic *β* cells during diabetes.

Increased superoxide production in the development and progression of diabetes causes the activation of several signal pathways involved in the pathogenesis of chronic complications. Oxidative stress activates cellular signaling pathways and transcription factors, including protein kinase C (PKC), c-Jun-N-terminal kinase (JNK), p38 mitogen-activated protein kinase (MAPK), and nuclear factor kappa-B (NF-*κ*B) [18]. Recently, we have shown that signal transduction systems of immune cells are involved in the development of diabetes in animals, with a special role played by the nuclear factor kappa B (NF-*κ*B) cascade [19]. Thus, we showed that the use of an inhibitor of the NF-*κ*B signaling pathway, as well as the thymic hormone thymulin, and a diet with antioxidants significantly reduced the immune imbalance in cells of mice with alloxan-induced diabetes [20].

In the present study, the efficacy of PRDX6 for reducing the damaging effects of alloxan-induced diabetes in mice was studied for the first time. Furthermore, taking into account the key role of the pancreatic *β* cell loss in the development of diabetes mellitus, we studied the effects of PRDX6 on the viability and functional activity of the RIN-m5F *β* cell line under conditions that simulate diabetes.

## 2. Materials and Methods

### 2.1. Animals, Diabetes Model, and Peroxiredoxin 6 Treatment

Six- to eight-week-old male BALB/c mice (22–25 g) were maintained under standard laboratory conditions (20–21°C, 10–14 h light/dark cycle, and 65% humidity), with food and water provided ad libitum. Standard food pellets contained a balanced diet of proteins, vitamins, and minerals according to the Code of Practice for the Housing and Care of Animals Used in Scientific Procedures [21]. Experimental procedures were approved by the Institutional Ethical Committee (approval #57, 30/12/2011), and the experiments were performed in accordance with the Guidelines for Ethical Conduct in the Care and Use of Animals. Mice were sacrificed using cervical dislocation and decapitated using a small animal guillotine with a sharp blade.

Severe diabetes was modelled using a single intraperitoneal injection of 500 mg/kg alloxan in 0.2 mL of physiological saline, and physiological saline was injected into the control mice. Experiments were performed 10 days after the injection, when the blood glucose was consistently greater than 18 mM, indicating that the mice were diabetic. The mortality rate of the parallel groups of diabetic mice was observed for 32 days. Blood glucose concentration was measured using a glucometer (Accu-Chek Performa Nano, Germany) and Test Strips (Accu-Chek Performa Solo, Germany). A drop of blood was obtained from the tip of the tail of fasting mice.

PRDX6 (20 mg/kg body weight in 0.1 mL saline) was applied intravenously directly before the onset of diabetes on the first day and repeatedly on the eighth day during diabetes development. Control mice received intravenously 0.1 mL of physiological saline. Previously, we found that after intravenous administration, PRDX6 retained the highest level in the blood for 10 minutes; then, its amount gradually decreased, but after 6 hours, about 30% of the administered PRDX6 remained in blood plasma [22]. So, it was proved that possible PRDX6 effects were caused by its presence in the animal tissues.

### 2.2. Isolation and Purification of the PRDX6

Genetic constructions encoding (human) PRDX6 enzymes were obtained and expressed earlier in E. coli BL21(DE3) cells [22]. Recombinant proteins harbored His-tag, so the enzymes were purified by affinity chromatography on Ni-NTA-agarose (Thermo Fisher Scientific, USA), according to the manufacturer's recommendations. The technique of protein isolation was described earlier [23]. According to electrophoresis in 12% SDS-PAAG, the purity of the obtained enzymes was at least 98%. PRDX6 in phosphate buffer (1.7 mM KH_2_PO_4_, 5.2 mM Na_2_HPO_4_, and 150 mM NaCl, pH 7.4) at a concentration of 10 mg/mL were stored at –20°C. No reduction of enzymatic activity was observed following 2 months of storage.

The ability of the PRDX6 to reduce hydrogen peroxide (H_2_O_2_) and tert-butyl-hydroperoxide (t-BOOH) was determined by Kang et al.'s method [24], with minor modifications. Peroxidase activity of recombinant PRDX6 was 230 nmol/min/mg of protein (measured with H_2_O_2_) and 100 nmol/min/mg of protein (measured with t-BOOH).

### 2.3. Blood Plasma

Plasma was isolated from the blood collected during decapitation of the mice. Blood samples were kept for 3–5 h at 4°С and then centrifuged at 200 × *g*; the supernatants were collected for cytokine assays. Splenic lymphocytes were isolated in Dulbecco's modified Eagle's medium (DMEM; Sigma, USA) containing 10 mM 4-(2-hydroxyethyl)-1-piperazineethanesulphonic acid solution, 100 *μ*g/mL streptomycin, and 10% fetal bovine serum. Erythrocytes were lysed in Tris-buffered ammonium chloride (0.01 M Tris-HCl, with 0.15 M NaCl, and 0.83% NH_4_Cl at 9 : 1, pH 7.2). After being washed, the samples were stored at a concentration of 1 × 10^8^ cells/mL in RPMI 1640 medium at −20°C.

### 2.4. Cytokine Measurements

Enzyme-linked immunosorbent assays (ELISAs) were used to determine concentrations of cytokines in blood plasma using ELISA development kits for mouse TNF-*α*, IL-5, IL-17, and IFN-*γ* (PeproTech, USA). Binding was visualised by adding 100 *μ*L of the 2,2′-azino-*bis*(3-ethylbenzthiazoline-6-sulphonic acid) green dye (Sigma), dissolved in 0.05 M citrate buffer (pH 4.0) with 0.01% hydrogen peroxide. Absorbance was measured at 405 nm using a Multiscan EX spectrophotometer (Thermo Electron Corporation, Vantaa, Finland).

### 2.5. Western Blotting Analysis

To prepare specimens, 1 × 10^8^ splenic cells were lysed using a lysis buffer containing 50 mM Tris-HCl (pH 7.4), 150 mM NaCl, 1% Triton X-100, and 5 mM ethylenediaminetetraacetic acid (Alfa Aesar, UK). The total protein concentration was determined using Bradford's method [25] (Sigma) following protein precipitation with acetone in an ice bath for 15 min. Next, proteins were diluted 1 : 1 with a solution containing 65.8 mM Tris-HCl, pH 6.8, 2.1% sodium dodecyl sulphate, 26.3% (*w*/*v*) glycerol, and 0.01% bromophenol blue, boiled for 5 min, and stored at 4°C. Proteins were resolved by 10% polyacrylamide gel electrophoresis using a protein MW marker (Thermo Scientific, USA) and then transferred onto a nitrocellulose membrane (GE Healthcare, Amersham, UK) in a transblot chamber. After being blocked with 5% (*w*/*v*) nonfat dry milk in Tris-buffered saline/Tween 20, the membranes were exposed for 2 h to the following antibodies raised against mouse proteins: a phospho-NF-*κ*B p65 (Ser 536) antibody (#3031, Cell Signaling Technology, Danvers, MA, USA), rabbit phosphoinhibitor of NF-*κ*B kinase (IKK*α*/*β*) antibody II (Ser 176/180) (Cell Signaling Technology), rabbit phospho-stress-activated protein kinase/c-Jun N-terminal kinase (SAPK/JNK) antibody to synthetic phospho-SAPK/JNK peptide (Cell Signaling Technology), and rabbit caspase-3 monoclonal antibody (Cell Signaling Technology). After being washed, the membranes were incubated for 1 h with an anti-rabbit biotinylated antibody (Jackson ImmunoResearch, West Grove, PA, USA), followed by incubation with peroxidase-conjugated streptavidin for 1 h. As a loading control, glyceraldehyde 3-phosphate dehydrogenase (GAPDH) was used, which was detected with a rabbit monoclonal antibody raised against a synthetic peptide corresponding to residues near the C-terminus of human GAPDH (Cell Signaling Technology). The ECL Plus chemiluminescent cocktail (Amersham/GE) was used to develop the blots according to the manufacturer's instructions. The developed blots were photographed using a TFX-35.WL transilluminator (Vilber Lourmat, France). Protein bands were quantified densitometrically using Image Studio Software, version 5.2 (LI-COR, NE, USA). Two-three independent experiments were performed for each protein using cells from different passages or splenocytes from individual animals. The obtained data were normalised to the corresponding loading control (GAPDH) and expressed in relative units.

### 2.6. Histology and Immunohistochemistry

The pancreases from different groups were fixed in 10% formalin, then embedded in paraffin, and 3 *μ*m sections were prepared and stained with haematoxylin and eosin (H&E) for histopathological examination. For the detection of insulin, the 3 *μ*m sections were dewaxed, rehydrated, and incubated with a peroxidase-blocking reagent (DAKO Cytomation, Fort Collins, CO, USA) to block endogenous peroxidase. Next, the slides were incubated with phosphate-buffered saline (PBS)+1% bovine serum albumin to block nonspecific binding. A rabbit anti-mouse insulin monoclonal antibody (Santa Cruz Biotechnology, Santa Cruz, CA, USA) was subsequently applied to the sections, followed by incubation with the LSAB™ system-HRP (DAKO Cytomation). The slides were stained with diaminobenzidine according to the manufacturer's instructions (DAKO Cytomation).

### 2.7. Quantification of *β* Cell Mass

Images of the sections were analysed using the ImageJ software (National Institutes of Health, USA, http://rsb.info.nih.gov/ij/) to measure insulin-positive areas and the total pancreas area. The *β* cell mass (mg) per pancreas was calculated by multiplying the relative insulin-positive area (the percentage of insulin-positive area over total pancreas area) by the pancreas weight as was reported earlier [26].

### 2.8. Culture of RIN-m5F Cells

Rat insulinoma RIN-m5F cells (Vertebrate Cell Collection, St. Petersburg, Russia) were grown in culture flasks in a medium, with a low glucose content (8.0 mM), consisting of a mixture of RPMI 1640 medium/DMEM (1 : 1), supplemented with 10% fetal calf serum (FCS), 100 *μ*g/mL penicillin, 100 *μ*g/mL streptomycin, and 50 *μ*g/mL gentamicin, at 37°C and 5% CO_2_. Cells were only used between passages 3 and 7 and were cultured for 24 h and washed. To induce apoptosis, 30 ng/mL TNF-*α* (Recombinant Murine TNF-*α*, PeproTech)+15 ng/mL IL-1*β* (Recombinant Murine IL-1*β*, PeproTech) were added. Recombinant PRDX6 (150 *μ*g/mL) was added 30 min before the addition of the cytokines. Cells were cultured for 24 h and washed before measuring the viability and signal proteins. In each independent experiment performed using separate passage, the measurements were provided for 9–12 replicates. The average values from four independent experiments were used to determine the significance of differences between groups. Cells incubated without the cytokines and PRDX6 were used as controls.

### 2.9. Measurement of ROS Using Carboxy-2′,7′-Dichlorodihydrofluorescein Diacetate (H_2_DCFDA)

RIN-m5F cells were cultured for 24 h in a 96-well plate (2.5 · ×10^4^ cells per well in 100 *μ*L) in DMEM and then washed with PBS. The cells were then incubated with carboxy-H_2_DCFDA (Invitrogen, USA; freshly prepared in sterile DMSO) at a final concentration of 2.5 *μ*M in the medium supplemented with 2% depleted FCS in the darkness for 1 h; then, PRDX6 and the cytokines were added, and the cells were incubated for another 1 h. Cells incubated in the absence of PRDX6 and cytokines were used as a control. The fluorescence was measured at an excitation of 485 nm and an emission of 535 nm using an Infinite 200 plate reader (Tecan, Austria), as described earlier [27].

### 2.10. Statistical Analysis

Statistical analysis was performed using the Statistica/Win 6.0 software (Tulsa, OK, USA). One-way analysis of variance, followed by Tukey's post hoc test, was performed to determine the significance of differences among groups. Values of *p* ≤ 0.05 were considered significant.

## 3. Results

### 3.1. Effects of PRDX6 on Mortality Rate and Plasma Glucose Levels in Diabetic Mice

**Figure 1:**
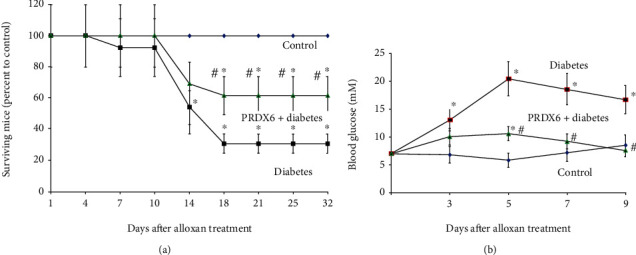
Effects of PRDX6 on the mortality rate of mice and on blood glucose in diabetic animals. (a) Time course of mortality in different groups of mice (mean ± standard deviation (SD) of 10 mice). (b) Time course of changes in plasma glucose in different groups of mice (mean ± standard deviation (SD) of 10 mice). ^∗^*p* < 0.05 vs. control; ^#^*p* < 0.05 vs. the diabetic group.

Three groups of mice were used (alloxan-treated, alloxan plus PRDX6-treated, and untreated age-matched controls), and each group consisted of 10 mice. It was revealed that the mortality rate following administration of a high dose of alloxan (500 mg/kg) achieved about 80% on the 32nd day after alloxan treatment (Figure 1(a)). The mortality rate of diabetic mice pretreated with PRDX6 was markedly lower compared to diabetic mice that did not receive PRDX6.

Another three mouse groups consisting of 10 mice per group (alloxan-treated, alloxan plus PRDX6-treated, and untreated age-matched controls) were observed for blood glucose. Blood glucose levels were measured for 9 days after the administration of alloxan (Figure 1(b)). It was demonstrated that on day 5 after the administration of alloxan, the average glucose level in the blood of the alloxan-treated mice exceeded 20 mM, while that in the mice treated with PRDX6 decreased to almost the control level (Figure 1(b)). Thus, the administration of PRDX6 inhibited the glycemia raise in mice with alloxan-induced diabetes. So, pathophysiological manifestations in mice treated with alloxan were significantly lowered by PRDX6 application.

### 3.2. Effects of PRDX6 on Spleen Cell Apoptosis in Diabetes Mice

**Figure 2:**
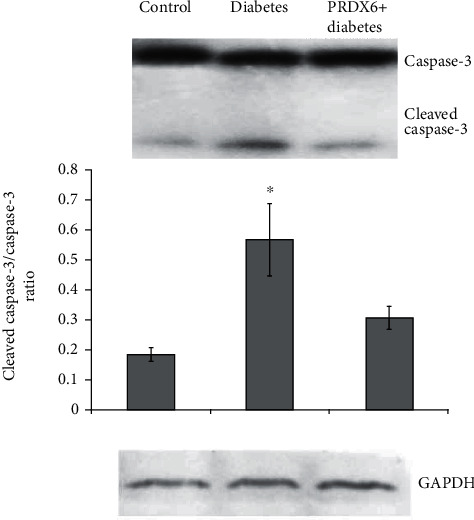
Expression of caspase-3 and cleaved caspase-3, determined using western blot analysis. Representative blots from three independent experiments are shown. Histograms show the protein levels normalised to those of GAPDH, used as a loading control, and to total forms of relative proteins and represent the average densitometry values for the blots from two experiments (mean ± SD). ^∗^*p* < 0.05 vs. control.

The level of apoptosis in splenocytes was assessed based on the ratio of activated/nonactivated caspase-3. A very sharp increase in the level of splenocytic apoptosis was observed in alloxan-induced diabetes (Figure 2). However, administration of PRDX6 to the diabetic mice completely normalised the ratio of activated/nonactivated caspase-3 in splenocytes, indicating a protective effect of PRDX6 in developed diabetes.

### 3.3. Effects of PRDX6 on Cytokine Levels in the Blood of Diabetic Mice

**Figure 3:**
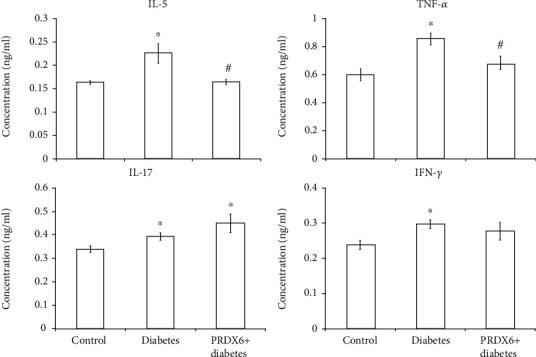
Effects of PRDX6 on plasma cytokine concentrations in diabetic mice. Each value is the mean ± SD for three mice; six measurements were performed for each individual mouse. ^∗^*p* < 0.05 vs. control; ^#^*p* < 0.05 vs. the diabetic group.

A study of the cytokine response demonstrated that in the mice with developed alloxan-induced diabetes, the concentrations of all measured cytokines in the plasma increased on the 10th day after the administration of alloxan, with the most marked increase observed in TNF-*α* and IL-5. The concentrations of IFN-*γ* and IL-17 also increased, although these changes were relatively small (Figure 3). The administration of PRDX6 reduced the peaks of TNF-*α* and IL-5 in the blood of diabetic mice. It is important to emphasise that the use of PRDX6 had no effect on the level of IL-17 in the blood of diabetic mice.

### 3.4. Effects of PRDX6 on the Pancreatic Islet Structure, Insulin Expression, and *β* Cell Mass in Diabetic Mice

**Figure 4:**
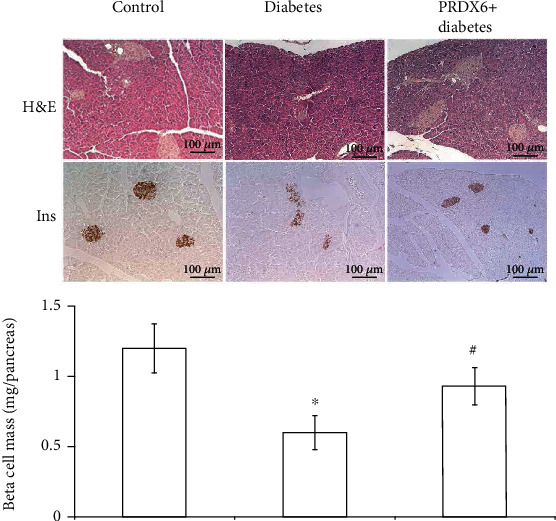
Effects of PRDX6 on the pancreas structure and *β* cell mass in diabetic mice. Representative images show islet histology using H&E staining and islet immunostaining for insulin in the pancreas of control and diabetic mice. The *β* cell mass is shown as the mean ± SD for 15 sections/pancreas from three individual mice. ^∗^*p* < 0.05 vs. control; ^#^*p* < 0.05 vs. the diabetic group.

To elucidate PRDX6 effects on the pancreas in diabetic mice, immunostaining for insulin was performed. Immunohistochemical examination of the pancreas revealed a reduction in the islet density in diabetic mice, and the residual *β* cells were severely disorganised (Figure 4). PRDX6 injections substantially restored the islet density in diabetic mice. Using sections from the control and diabetic pancreases, we quantitatively evaluated the *β* cell mass. As shown in Figure 4, the number of *β* cells in the diabetic pancreas was significantly smaller than that in the control pancreas. Treatment with PRDX6 markedly increased the *β* cell numbers, thereby protecting the islet structure. This finding was consistent with the effects of PRDX6 on the cytokine profile and cell apoptosis. The data demonstrated the expected destruction of pancreatic *β* cells in advanced diabetes and indicated that PRDX6 markedly increased the *β* cell mass, thus supporting a protective function of the antioxidant enzyme. Moreover, immunostaining confirmed the protective effect of PRDX6 on the immunity of mice with alloxan-induced diabetes.

### 3.5. Effects of Cytokines and PRDX6 on RIN-m5F *β* Cells

RIN-m5F *β* cells were cultured under adverse conditions (in the presence of proinflammatory cytokines), and the viability of these cells was studied with or without the PRDX6 addition. To elucidate the molecular mechanisms of the protective effects of PRDX6, the level of apoptosis and the activity of the NF-*κ*B and SAPK/JNK signaling cascades were determined in RIN-m5F *β* cells. In addition, the PRDX6 antioxidant activity was tested using a carboxy-H_2_DCFDA probe.

**Figure 5:**
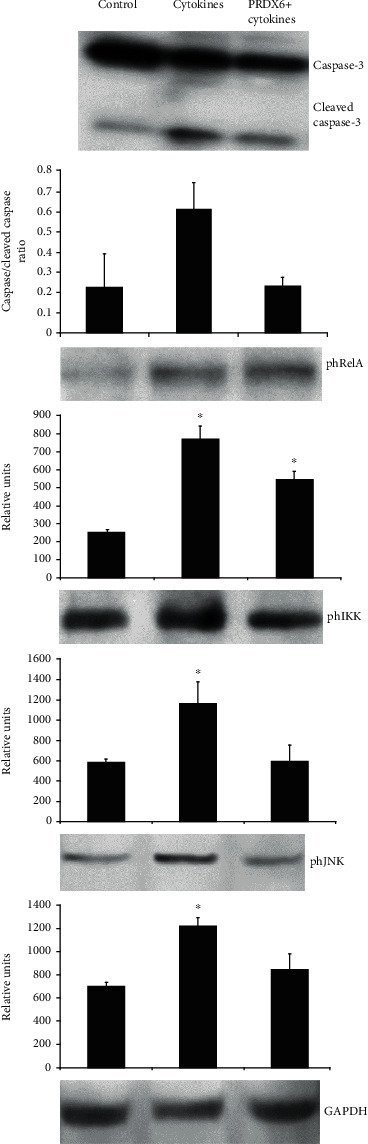
Effects of PRDX6 on the activation of caspase-3, NF-*κ*B, IKK, and JNK pathways in RIN-m5F *β* cells cultured with proinflammatory cytokines. Blot images are shown from a single representative experiment. Histograms show the protein levels (in relative units) normalised to those of GAPDH, used as a loading control, and to total forms of relative proteins, representing the average densitometry values for blots from two-three experiments with cells from different passages (mean ± SD). ^∗^*p* < 0.05 vs. control.

The level of apoptosis in RIN-m5F *β* cells was assessed by measuring the ratio of activated to nonactivated form of caspase-3 (Figure 5). It was demonstrated that the addition of the mixture of cytokines (TNF-*α* and IL-1*β*) to the cell culture medium led to a significant increase in the level of apoptosis. At the same time, the presence of PRDX6 in the medium completely eliminated the toxic effect of the cytokines, reducing the ratio of the activated to nonactivated form of caspase-3.

The activity of the NF-*κ*B signaling cascade was assessed based on the level of RelA/p65 protein phosphorylation at the Ser 536 residue (Figure 5) and IKK*α*/*β* activation. It was shown that in the cells incubated with the proinflammatory cytokines, the phosphorylation of the RelA/p65 protein at Ser 536 increased more than twofold. It is important to note that the addition of PRDX6 under these conditions had almost no effect on the phosphorylation of RelA/p65. On the other hand, incubation of RIN-m5F cells with the cytokines led to increased phosphorylation of IKK*α*/*β*. At the same time, PRDX6 completely alleviated the cytokine-induced IKK*α*/*β* activation. Thus, PRDX6 decreased the activation of the canonical NF-*κ*B pathway in RIN-m5F *β* cells under conditions of oxidative stress caused by proinflammatory cytokines.

An even more profound protective effect of PRDX6 was revealed by studying the JNK activity in RIN-m5F cells incubated with the proinflammatory cytokines. Indeed, the addition of the cytokines to the RIN-m5F cell culture medium led to the activation of the JNK signaling cascade, while the addition of PRDX6 completely blocked the cytokine-induced JNK activation in *β* cells.

**Figure 6:**
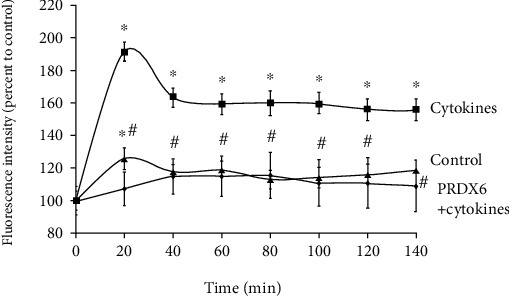
Effect of PRDX6 on the ROS levels in RIN-m5F cells in the presence of proinflammatory cytokines. Cells were incubated for 1 h in the presence of the fluorescent dye carboxy-H_2_DCFDA, with or without cytokines (TNF-*α*+IL-1*β*) and PRDX6 (150 *μ*g/mL). Each value presents the mean green fluorescence intensity from 9–12 repeats (as a percentage of control). ^∗^*p* < 0.01 vs. control; ^#^*p* < 0.05 vs. the cytokine treatment group.

Furthermore, the antioxidant efficiency of PRDX6 was also tested in vitro using RIN-m5F cells. As expected, the addition of the proinflammatory cytokine mixture to the cultural medium produced a sharp increase in the ROS content in these cells. The results also showed that the addition of PRDX6 significantly reduced the level of ROS in RIN-m5F cells cultured in the presence of TNF-*α* and IL-1*β* (Figure 6).

Thus, we obtained evidence that the protective effects of PRDX6 in T1D are related to its antioxidant activity. Interestingly, using another model of oxidative stress *in vivo*, we showed that the preliminary PRDX6 treatment reduced the level of malonic aldehyde in the ischemia-reperfusion kidney [22].

## 4. Discussion

Reduced antioxidant activity and increased oxidative stress are among the intrinsic characteristics of T1D mellitus, both in patients and in animal diabetes models. T1D is provoked by the destruction of pancreatic *β* cells, which is mediated via an autoimmune mechanism and consequent inflammatory processes. Numerous inflammatory cytokines and ROS, which are produced during development of diabetes, have been proposed to play an important role in *β* cell destruction. For example, ROS can penetrate through the cell membrane and cause damage to *β* cells of the pancreas [28]. Possible causes of T1D are genetic or involve chemical, immune, or virus-associated damage to insulin-producing *β* cells. Regardless of the cause, a common pathway may exist that leads to the destruction of *β* cells.

Animal models play an important role in the development of present concepts concerning T1D pathogenesis and therapeutic approaches. In the present study, we modelled a severe and rapidly developing form of T1D using a large dose of alloxan. Indeed, worse blood glucose control in patients with diabetes is generally associated with a more rapidly progressing disease and a wider range of complications. Using this model, we demonstrated the beneficial effect of PRDX6 on the T1D pathology in terms of the plasma glucose level, plasma cytokine profile, and splenic cell apoptosis. Moreover, using histological and immunohistochemical assays, we studied the pancreatic islet structure and insulin expression in *β* cells after treatment of diabetic mice with PRDX6.

In addition, to elucidate the molecular mechanisms of PRDX6 protective activity, rat insulinoma RIN-m5F сells were cultured with cytokines (TNF-*α* and IL-1*β*) and used as an in vitro diabetic model to measure the ROS secretion, *β* cell apoptosis, and the activity of the NF-*κ*B and JNK signaling pathways.

Among proinflammatory plasma cytokines, TNF-*α*, IFN-*γ*, IL-5, and IL-17 were shown to increase in alloxan-induced diabetic mice, which may indicate the activation of T helper 1 (Th1) cells, producing TNF-*α* and IFN-*γ*; Th2 cells, producing IL-5; and Th17 cells, secreting IL-17 [29]. The treatment with PRDX6 reduced the plasma TNF-*α* and IL-5 levels but showed no significant effects on the plasma concentrations of IFN-*γ* and IL-17 in diabetic mice. Apparently, this can be explained by the fact that the use of various drugs to reduce the pathological effects of diabetes can be more or less effective, depending on the stage and severity of the disease. Indeed, it has earlier been demonstrated that antidiabetic approaches are more effective in prediabetic mice than in mice with advanced diabetes [20]. In addition, IFN-*γ* is known to inhibit Th17 cells, and the role of Th17 cells in T1D remains largely unknown [30].

More clear evidence of the protective activity of PRDX6 was obtained by the measurement of the blood glucose level, as well as by the assessment of the level of apoptosis of splenocytes. Indeed, administration of a large dose of alloxan caused a sharp increase in the blood glucose level in diabetic mice within 5 days after the administration, and glucose remained at a high level throughout the experiment. In the mice treated with PRDX6, a slight increase in plasma glucose was observed on day 5, but by the end of the experiment, the plasma glucose level was fully normalised. These observations are in agreement with the data of our recent study showing the protective effects of PRDX6 on RIN-m5F сells cultured with high glucose concentrations [31]. It should be emphasised that according to our previous study, PRDX6 itself did not cause changes in either the activity of the NF-*κ*B and JNK signaling cascades or the level of apoptosis of *β* cells, estimated by the ratio of activated to nonactivated form of caspase-3 [31]. Thus, preliminary results indicate the nontoxicity of the PRDX6 recombinant protein.

It is generally known that the pathogenic effect of hyperglycemia is mediated, to a significant extent, via increased production of ROS. Therefore, it seems quite possible that PRDX6 may actually reduce the oxidative stress in diabetic mice, as was shown in the present in vitro study using RIN-5mF cells. In addition, alloxan-induced diabetes is a rapidly developing severe form of diabetes, which may be more dependent on oxidative stress than other forms. Consistent with our results, another study demonstrated that acute elevation of glucose resulted in a more specific triggering effect on oxidative stress than did chronic sustained hyperglycemia [32].

It is commonly known that *β* cell death in T1D involves necrosis and apoptosis [33]. One of the immunocytochemical markers for apoptosis is cleaved caspase-3. The caspase-3 protein is a member of the cysteine-aspartic acid protease (caspase) family and plays a central role in the execution phase of cell apoptosis [34]. The ubiquitously distributed caspase-3 is the main effector of the apoptotic cascade within cells and is activated through cleavage [35].

In the present study, we demonstrated that both *β* cells in vitro and splenic cells in vivo underwent apoptosis in situations modelling T1D, and the treatment with PRDX6 substantially reduced the diabetogenic apoptosis, thus indicating a protective effect of PRDX6. In addition, the loss of the *β* cell mass, observed in mice with alloxan-induced diabetes and mediated by the activation of proapoptotic signaling events, is increasingly recognised as the causal and committed stage in the development of T1D mellitus. Thus, our data suggest that this stage may also be alleviated by PRDX6 administration.

The recombinant PRDX6 can affect the level of ROS in animals, inhibiting the development of oxidative stress and normalising the redox status of cells. However, the question arises: how does recombinant PRDX6 located in the extracellular space neutralise ROS in cells into which it does not penetrate? It is known that hydroperoxides, in addition to passive diffusion through the cell membrane, are actively transported to the intercellular space using aquaporins [36]. Probably, being in the extracellular space, recombinant PRDX6 can participate in the elimination of peroxides formed in the intercellular space and released from the cells by the aquaporins.

Earlier, it was shown that PRDX6 can affect the level of NF-*κ*B via the TLR4/NF-*κ*B signaling pathway [37]. The authors showed that during ischemic damage of the brain, PRDX6 released during the destruction of cells can act as an endogenous ligand for the TLR4 receptor. The interaction of PRDX6 with TLR4 triggers a cascade of processes in which NF-*κ*B plays a major role, resulting in an emergency cell repair and suppression of apoptosis [38]. It is possible that intravenous application of the recombinant PRDX6 before exposure to alloxan can lead to a preconditioning effect, inducing the mechanisms of repair and antioxidant response through stimulation of the TLR4/NF-*κ*B signaling pathway in the cells. Therefore, exposure to alloxan does not lead to a synergistic increase in NF-*κ*B expression.

In addition to the peroxidase activity of PRDX6, a Ca^2+^-dependent phospholipase A2 activity (aiPLA2) of PRDX6 was also shown, which normally manifests itself only under acidic conditions and plays an important role in the metabolism of phospholipids and the transmission of intracellular/intercellular signals [39]. Interestingly, with an excess of ROS, peroxidase cysteine center of PRDX6 is oxidized, which leads to a significant increase in the Ca^2+^-dependent phospholipase A2 activity (aiPLA2) [39]. In addition, regardless of the oxidation of the peroxidase center, an increase in the phospholipase activity (more than 10 times) of PRDX6 is observed after specific phosphorylation of the Thr177 residue by mitogen-activated protein kinases (MAPKs) (ERK2, p38*γ*, and p38*δ*) [40]. Accordingly, with the induction of the aiPLA2-activity of PRDX6, there is an increase in the level of lysophospholipids and fatty acids, which serve as secondary messengers both in normal and in pathologies. It has been shown that the phospholipase activity of PRDX6 stimulates signaling pathways (p38, PI3K/Akt) and also promotes the formation of arachidonic acid, which, in turn, affects the activity of Src (SFK) kinases, stimulating cell growth and division [41].

We have shown earlier that in slowly developing diabetes, induced by a small dose of alloxan, the most significant activation of several signaling cascades, including the interferon regulatory factor 3, Toll-like receptor 4, and NF-*κ*B pathways, as well as an increase in the expression of heat shock protein 70 in splenic cells, was observed only at the prediabetes stage but not in advanced diabetes [20]. However, the JNK pathway was an exception in this regard, being activated more significantly at the stage of advanced diabetes. In the present study, we demonstrated that the JNK pathway was profoundly activated in *β* cells by proinflammatory cytokines and oxidative stress. This does not contradict the findings that demonstrated the role of both NF-*κ*B and JNK signaling in rat *β* cell death that is induced by proinflammatory cytokines [42]. Interestingly, PRDX6 downregulated the JNK activity in *β* cells exposed to TNF-*α* and IL-1*β*, suggesting that oxidative stress and subsequent activation of the JNK pathway could be involved in the pathogenesis of T1D. This study investigated whether the NF-*κ*B and JNK pathways are involved in the protective effect of exogenous PRX6 against *β* cell destruction induced by alloxan treatment in mice or by proinflammatory cytokines in RIN-m5F cells. We demonstrated that the decrease in the ROS levels in RIN-m5F cells was accompanied by improvement of activity of caspase-3, IKK, and JNK pathways in RIN-m5F *β* cells cultured with proinflammatory cytokines. In addition, PRDX6 administrated to diabetic mice partially improved the plasma cytokine profile and tended to restore the pancreas structure and *β* cell mass in diabetic mice.

The importance of the NF-*κ*B pathway was demonstrated in both T1D and T2D, due to its role in inflammatory responses [43]. We have previously shown involvement of NF-*κ*B, namely, RelA/p65, in the protection of pancreatic *β* cells in alloxan-induced diabetic mice, using an inhibitor of this cascade (IKK inhibitor XII) [20]. It was demonstrated that knockdown of PRDX6 increased susceptibility of RIN-m5F cells to the deleterious effects of proinflammatory cytokines and to oxidative stress [44]. These results show that among the PRDXs significantly expressed in RIN-m5F cells, only PRDX6 is modulated by the proinflammatory cytokines, and the PRDX6 downregulation depends on the calpain, proteasome systems, and JNK signaling [44]. Moreover, the link between PRX6 and NF-*κ*B, which is one of the most prominent redox-regulated proinflammatory regulators, has been previously observed in hypoxic mouse hippocampal cells [45]. Similarly, PRDX6 expression is inversely correlated with NF-*κ*B during *Clonorchis sinensis* infection [46]. Interestingly, NF-*κ*B activation is crucial for maturation and activation of immune cells, but in *β* cells, it has mostly a deleterious effect [47]. So, understanding the innate inflammation and mechanisms by which *β* cell susceptibility to proinflammatory cytokines is potentiated or mitigated offers important insight into T1D progression and avenues for therapeutic intervention [12].

In conclusion, PRDX6 treatment reduced cell apoptosis in both *in vitro* and *in vivo* models of T1D. Thus, PRDX6 protected RIN-5mF cells against cytokine-induced cytotoxicity in vitro, and it also prevented T1D, normalised blood glucose, lowered mortality rate, and restored the pancreatic islet structure in vivo. Moreover, PRDX6 normalised the activity of the NF-*κ*B and JNK pathways in *β* cells cultured with proinflammatory cytokines and protected these cells from ROS. Therefore, there is a prospect for therapeutic application of PRDX6 to alleviate or even prevent *β* cell apoptosis in T1D.

## 5. Conclusion

The study demonstrates that administration of recombinant, exogenous PRDX6 reduces impact of the alloxan toxicity on pancreatic *β* cells in mice, improving animal mortality and lowering the serum glucose level. The beneficial effects of recombinant PRDX6 treatment are associated with reduced levels of inflammatory cytokines in vivo. Meanwhile, in the rat insulinoma cell line, the recombinant PRDX6 attenuated effect of exogenous proinflammatory cytokines on the ROS production and activation of NF-*κ*B and JNK signaling pathways. In summary, the manuscript provides novel observations, which have clear implications in understanding the cytotoxic mechanism underlying death of *β* cells in DM1 and in forming potential therapeutic approaches.

## Data Availability

The data used to support the findings of this study are included within the article.
